# 

**Published:** 1883-07-20

**Authors:** 


☞ MANY of our subscribers are in arrears for subscription. Friends, let us hear from you at once, and oblige,

R. C. WORD, *Managing Editor.*

### *EDITORIAL NOTICES.*

☞ Receipts for subscription will appear in our next.

THE CODE AT THE AMERICAN MEDICAL ASSOCIATION.—The Judicial Council of th e Association required the members on registering to indicate their acceptance of the Code, thus eliminating from the discussions all trouble or disturbance upon the subject.

AT a late meeting of the American Medical Association, a paper signed by Drs. Gross, Flint and Wendell Holmes was read, proposing to ask an appropriation from Congress to provide a fire-proof building for the Museum and Library of the Surgeon-General's office at Washington. The Association accordingly adopted a resolution asking $10,000 for that purpose.

INEBRIATE ASYLUM.—The Georgia Legislature has now under consideration a bill for the establishment of an Inebriate Asylum in the State. This is in response to a memorial sent up by the State Medical Association. The prospect of the passage of the bill is regarded as very hopeful.

Such an Institution is certainly needed, and accords well with the growing interest of public sentiment in respect to the evils of intemperance and the necessity of legal interposition to suppress and remedy them.

### *PAMPHLETS RECEIVED.*

THE Cornell University Register, for 1882 and 1883.

ONTARIO MEDICAL ASSOCIATION.—Report of the committee on Ophthalmology, 1882.

HANDBOOK of Medical Electricity with a description of a new medical battery, by A. M. Rosebrugh, M. D., Surgeon to the Toronto Eye and Ear Dispensary; Member of the International Ophthalmological and Otological Societies, etc., etc.

PEMPHIGUS, and the diseases liable to be mistaken for it, by George H. Rohe, M. D., Professor of Hygiene and Clinical Dermatology, College of Physicians and Surgeons, Baltimore: Member of the American Dermatological Association, etc.

HINTS on the treatment of some Parasitic Skin Diseases, by George H. Rohe, M. D., Professor of Hygiene and Clinical Dermatology, College of Physicians and Surgeons, Baltimore; Member of the American Dermatological Association, etc.

OFFICERS, Rules and Schedule of Premiums of the Virginia State Agricultural Society for the twenty-third Annual Fair, on October 31 and November 1 and 2, 1883, at the Fair grounds, Richmond.

THE Treatment of the various forms of Acene, by George H. Rohe, M. D., Professor of Hygiene and Clinical Dermatology, College of Physicians and Surgeons, Baltimore; Member of the American Dermatological Association; of the American Public Health Association, etc.

